# Bilateral dilation of the urinary tract due to iliopsoas pyomyositis: a case report

**DOI:** 10.1186/1752-1947-5-195

**Published:** 2011-05-20

**Authors:** Aristotelis Tsiakalos, Sarah P Georgiadou, Ioannis Anastasiou, Nikolaos V Sipsas, Athanasios Tzioufas

**Affiliations:** 1 Department of Pathophysiology, Medical School, National and Kapodistrian University of Athens, Athens, Greece; 2 Department of Urology, Medical School, National and Kapodistrian University of Athens, Athens, Greece

## Abstract

**Introduction:**

Pyomyositis is an acute bacterial infection of the skeletal muscles that arises from hematogenous spread and is caused predominantly by Gram-positive cocci.

**Case presentation:**

We report a case of iliopsoas pyomyositis in a 25-year-old Greek Caucasian woman with a history of intravenous drug use. Her condition was complicated by bilateral dilation of the ureters and renal calyces as a result of mechanical pressure from inflammation and edema of the involved muscle. The patient did not present aggravation of renal function and was treated successfully solely with intravenous antibiotics, without surgical intervention. This is the first case report describing iliopsoas pyomyositis with reversible bilateral dilation of the urinary tract that was treated successfully with intravenous antibiotics, without surgical intervention.

**Conclusion:**

We present the first described case of iliopsoas pyomyositis with reversible bilateral hydroureteronephrosis that was treated successfully with intravenous antibiotics, without the necessity of surgical intervention. To our knowledge, this is the first report of its kind in the literature regarding an unexpected event in the course of treating a patient with iliopsoas pyomyositis, and it should be of particular interest to different clinical medical specialties such as internal medicine, infectious disease and urology.

## Introduction

Pyomyositis is an acute bacterial infection of the skeletal muscles that arises from hematogenous spread [1,2]. Most cases of pyomyositis occur in the tropics, although it has been recognized as an emerging phenomenon in temperate climates [3-5]. Approximately 40% of patients with pyomyositis who live in areas with temperate climates lack any relevant underlying disease. The remaining cases have predisposing conditions such as intravenous (IV) drug use (IVDU), trauma, immunosuppressive illnesses (for example, diabetes mellitus, malignancy, cirrhosis, renal insufficiency, human immunodeficiency virus (HIV) infection) and administration of immunosuppressive agents. *Staphylococcus aureus *is responsible for 95% of the cases in tropical areas and up to 75% of the cases in temperate zones. Group A streptococci are the second most common pathogen. Other rare causes of pyomyositis include various streptococci (groups B, C and G), *Streptococcus pneumoniae*, enterobacteriaceae, anaerobes, mycobacteria and fungi. The most frequent sites of involvement are the large muscles of the lower extremities (for example, the quadriceps, calves and the gluteus group) and the upper arm muscles, although a variety of other muscles can be involved [1-5].

In iliopsoas pyomyositis, the source of infection is usually an adjacent structure, but rarely primary abscesses develop via the hematogenous route. In this report, we present a rare case of left iliopsoas pyomyositis complicated by bilateral dilation of the urinary tract caused by mechanical pressure from inflammation and edema of the involved muscle.

## Case report

A 25-year-old Greek Caucasian woman with a history of IVDU and chronic hepatitis C virus was admitted to our department because of a four-day history of fever (39.0°C) associated with left lower quadrant abdominal pain. She recalled an injection of heroin into her left groin seven days prior to her presentation to our hospital. At the time of presentation to our hospital, the patient's temperature was 39.1°C, her heart rate was 118 beats/minute, her respiratory rate was 20 breaths/minute, her blood pressure was 115/70 mmHg and her oxygen saturation was 97%. Her physical examination revealed sensitivity to palpation of the left quadrant abdomen without signs of peritoneal irritation. The rest of the physical examination was unremarkable.

Her laboratory work-up at the time of admission showed normochromic normocytic anemia with a hematocrit level of 31.3% and a hemoglobin level of 10.6 g/dl. Her white blood cell count was 15×10^9^/l with a neutrophil count of 12.6×10^9^/l. Her serum urea was 14 mg/dl, creatinine was 0.6 mg/dl, glucose was 67 mg/dl and lactate dehydrogenase was 387 U/l. Her C-reactive protein level and erythrocyte sedimentation rate (ESR) were significantly elevated at 152 mg/dl and 110 mm/hour, respectively. Her liver function tests were as follows: total bilirubin, 2.24 mg/dl; aspartate aminotransferase, 26 U/l (normal, 40 U/l); alanine aminotransferase, 20 U/l (normal, 40 U/l); alkaline phosphatase, 482 U/l (normal, 280 U/l); and γ-glutamyl transpeptidase 136 U/l (normal, 32 U/l). Moreover, her serology was negative for HIV and hepatitis B virus. The results of urinalysis, electrocardiography and chest X-ray were normal.

**Figure 1:**
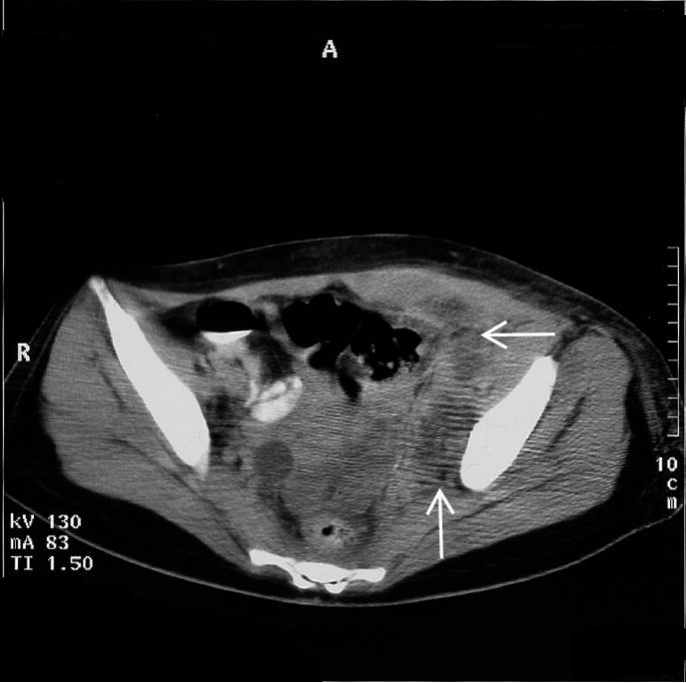
**Computed tomographic scan of the abdomen showing multiple small abscesses in the left iliopsoas (arrows) that established the diagnosis of pyomyositis**.

**Figure 2:**
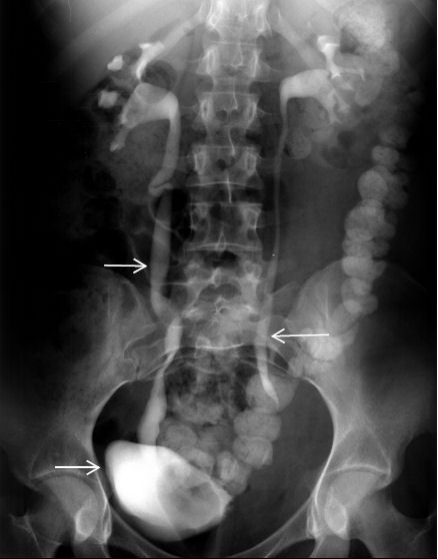
**Intravenous pyelogram showing bilateral dilation of the ureters and renal calyces, particularly on the right side, as well as a significant displacement of the urinary bladder**. The test was performed one week after the initiation of intravenous antibiotic therapy.

Computed tomography (CT) of her abdomen (Figure 1, arrows) revealed multiple small abscesses in the left iliopsoas, and as a result the diagnosis of pyomyositis was established. Blood was drawn for multiple cultures, and treatment with IV antibiotics (ampicillin/sulbactam and vancomycin) was started. Serial blood and tissue cultures obtained after a CT-guided needle biopsy were negative, and the patient continued on the same antibiotic regimen. One week later follow-up CT disclosed bilateral dilation of the ureters and renal calyces, particularly on the right side. IV pyelography confirmed the above findings and also showed a significant displacement of the urinary bladder (Figure 2, arrows).

The patient maintained efficient diuresis and normal renal function. Thus, no surgical intervention was performed. She remained hospitalized for one month receiving IV antibiotic therapy, and the follow-up IV pyelography and CT scan were both negative for dilations. She was discharged in good health with normalization of her biochemical and hematological parameters.

## Discussion

Pyomyositis pathogenesis involves transient bacteremia in the setting of muscle injury. Bacteremia without concurrent muscle damage is unlikely to cause myositis, since healthy muscle is quite resistant to infections. The large muscles of the lower extremities are most commonly affected, perhaps as a result of strenuous exercise or trauma leading to muscle injury [1,2]. However, any skeletal muscle group may be involved. Some studies have reported that primary psoas abscesses occur more frequently in younger patients, presumably because of the higher rates of IVDU and HIV infection within this age group. Other patient populations at high risk for psoas abscesses include patients with diabetes, older adults, people who are immunocompromised and alcoholics.

There is an association between IVDU and pyomyositis. This is thought to be due to impaired cellular and humoral immunity as well as abnormal skin bacterial colonization, along with the repeated introduction of non-sterile fluids into the body. In our case, the patient reported an injection of heroin into her left groin seven days before her hospital admission. Patients such as the one described here are therefore at risk of serious medical conditions not often seen in the general population [6-9].

Pyomyositis presents in three stages. Initially, there is myositis and muscle edema, but no abscess formation. At this stage, the condition may be cured by initiating appropriate antibiotic therapy. Stage 2, the suppurative phase, is characterized by abscess formation. The clinical presentation in stage 3 is complicated by systemic toxicity. Treatment in the latter stages of pyomyositis requires surgical incision and drainage as well as appropriate antibiotics [1,2].

Our patient, even though she was diagnosed with stage 2 pyomyositis, was treated successfully solely with antibiotics, without the necessity of surgical intervention. Furthermore, she progressively developed asymptomatic bilateral dilation of the urinary tract which was not accompanied by aggravation of renal function. The reason for her normal renal function is probably that hydronephrosis was caused by partial obstruction and because the obstruction remained for a short time. Hydronephrosis with asymptomatic obstruction is a normal finding in pregnant women, in whom the condition is caused by hormonal and mechanical factors [10]. The radiological findings of the IV pyelography in our patient (Figure 2) were attributed to a domino-like mechanical phenomenon with the following sequence: edema of the left iliopsoas as a result of inflammation of the area caused proximal dilation of the left ureter and displacement of the urinary bladder to the right side, which in turn led to proximal and distal dilation of the right ureter and right calyx as a result of mechanical pressure.

## Conclusions

In conclusion, we present the first described case of iliopsoas pyomyositis with reversible bilateral dilation of the urinary tract that was treated successfully with IV antibiotics, without surgical intervention. To our knowledge, this is the first report of its kind in the literature regarding an unexpected event in the course of treating a patient with iliopsoas pyomyositis, and it is an original case report of interest to different clinical medical specialties, such as internal medicine, infectious disease and urology.

## Consent

Written informed consent was obtained from the patient for publication of this case report and any accompanying images. A copy of the written consent is available for review by the Editor-in-Chief of this journal.

## Competing interests

The authors declare that they have no competing interests.

## Authors' contributions

AT and SPG wrote the manuscript. IA performed the urological evaluation and the interpretation of the intravenous pyelograms. NVS and AT corrected the manuscript. All authors read and approved the final manuscript.
